# Online symptom checker diagnostic and triage accuracy for HIV and hepatitis C

**DOI:** 10.1017/S0950268819000268

**Published:** 2019-03-04

**Authors:** A.C. Berry, B.D. Cash, B. Wang, M.S. Mulekar, A.B. Van Haneghan, K. Yuquimpo, A. Swaney, M. C. Marshall, W.K. Green

**Affiliations:** 1 Division of Gastroenterology, Larkin Community Hospital, Miami, FL, USA; 2 Division of Gastroenterology, Hepatology, and Nutrition, University of Texas Health Science Center at Houston, Houston, TX, USA; 3 Department of Mathematics and Statistics, University of South Alabama, Mobile, AL, USA; 4 University of South Alabama College of Medicine, Mobile, AL, USA; 5 Kansas City University of Medicine and Biosciences, Kansas City, MO, USA; 6 University of Alabama at Birmingham, Birmingham, AL, USA; 7 Division of Infectious Diseases, University of South Alabama, Mobile, AL, USA

**Keywords:** Emergency room, hepatitis C, HIV, symptom checker, technology

## Abstract

We sought to address the prior limitations of symptom checker accuracy by analysing the diagnostic and triage feasibility of online symptom checkers using a consecutive series of real-life emergency department (ED) patient encounters, and addressing a complex patient population – those with hepatitis C or HIV. We aimed to study the diagnostic and triage accuracy of these symptom checkers in relation to an emergency room physician-determined diagnosis. An ED retrospective analysis was performed on 8363 consecutive adult patients. Eligible patients included: 90 HIV, 67 hepatitis C, 11 both HIV and hepatitis C. Five online symptom checkers were utilised for diagnosis (Mayo Clinic, WebMD, Symptomate, Symcat, Isabel), three with triage capabilities. Symptom checker output was compared with ED physician-determined diagnosis data in regards to diagnostic accuracy and differential diagnosis listing, along with triage advice. All symptom checkers, whether for combined HIV and hepatitis C, HIV alone or hepatitis C alone had poor diagnostic accuracy in regards to Top1 (<20%), Top3 (<35%), Top10 (<40%), Listed at All (<45%). Significant variations existed for each individual symptom checker, as some appeared more accurate for listing the diagnosis in the top of the differential, *vs.* others more apt to list the diagnosis at all. In regards to ED triage data, a significantly higher percentage of hepatitis C patients (59.7%; 40/67) were found to have an initial diagnosis with emergent criteria than HIV patients (35.6%; 32/90). Symptom checker diagnostic capabilities are quite inferior to physician diagnostic capabilities. Complex patients such as those with HIV or hepatitis C may carry a more specific differential diagnosis, warranting symptom checkers to have diagnostic algorithms accounting for such complexity. Symptom checkers carry the potential for real-time epidemiologic monitoring of patient symptoms, as symptom entries and subsequent symptom checker diagnosis could allow health officials a means to track illnesses in specific patient populations and geographic regions. In order to do this, accurate and reliable symptom checkers are warranted.

## Short report

The number of emergency department (ED) visits has increased substantially over recent years, with concern for long wait times, decreased efficiency and patient satisfaction, provider fatigue, and drain of healthcare resources [1, 2]. Many patients presenting end up not warranting emergent care and thus provide additional strain on the limited ED resources [1, 2]. There remains a constant pursuit to better delineate emergent from non-emergent care. Today, many patients with HIV and hepatitis C are surviving longer and learning to manage their chronic diseases. A significant portion of HIV patients are prescribed complex medications and carry the risk of medication adverse profile, complicating their clinical presentation. Patients with HIV or hepatitis C carry the potential to present with symptoms or complaints forcing the provider to broaden or adjust his or her differential diagnosis or triage level of care. A simple complaint may warrant a whole different diagnostic algorithm compared with an immunocompetant individual.

Online symptom checkers are applications designed for users to provide a means to input patient demographics and symptoms into a predetermined computer algorithm in hopes of reaching a differential diagnosis and appropriate venue for patient care. They not only provide a way to simultaneously reach worldwide populations in a cost-effective manner, but they adopt the societal demand for on-demand electronic healthcare, as roughly 75% of International Web users search for health information online [3]. Limited analysis to date has focused on symptom checker diagnostic and triage accuracy using predetermined academic clinical vignettes, with varying results depending on disease severity, commonality and subject matter, and tremendous variability between analysed symptom checkers [4, 5]. Original studies have only analysed broad clinical vignettes without studying differential diagnosis capabilities in a complex, immunocompromised population [4, 5]. Critiques of these original studies have called for consecutive, complex, real-patient cases with well-validated diagnosis as a criterion paramount for head-to-head performance of symptom checkers and physicians [6, 7]. We address the aforementioned limitations by analysing the feasibility of online symptom checkers using a consecutive series of real-life ED patient encounters, and addressing a complex patient population – those with HIV or hepatitis C – to see whether online symptom checkers are reliable diagnostic and triage tools for this complex, and ageing, patient population.

An ED retrospective analysis from September 2013 to September 2015 for a 10-day consecutive block each month was performed on 8363 adult patients, searching for ‘HIV’, ‘Hepatitis C’, ‘Hep C’, ‘HCV’. IRB approval was obtained. No distinction of laboratory data, treatment regimen or active disease state was made. Patients who came as a direct transfer or unable to consciously make a decision (i.e. encephalopathy) were excluded. Eligible patients included: 90 HIV, 67 hepatitis C, 11 both HIV and hepatitis C. Five online symptom checkers were utilised for diagnosis (Mayo Clinic, WebMD, Symptomate, Symcat, Isabel), three with triage capabilities (Symptomate, Symcat, Isabel). Patient demographics, medical history and presenting symptoms were retrospectively entered into each checker by study team using the ED physician patient note. Further data regarding patient ED visit was recorded. Patient admission status was not further delineated.

Symptom checker output was compared with ED data in regards to diagnostic accuracy and differential diagnosis listing. Whether the diagnosis was recorded at all, in the Top1, Top3, Top10 and mean rank of differential diagnosis was documented. The gold standard for diagnostic accuracy remains the physician, as it is the cornerstone of the profession, as all comparisons are based on the physician-determined diagnosis. Data analysis compared the physician-determined top diagnosis with the listing found in the symptom checker, yielding a percentage representing how often the physician-determined diagnosis was listed by the symptom checkers, be it Top1, Top3, Top10 and mean rank. Triage advice (emergent: go to ED and see physician now; or non-emergent: see a provider next few days) was compared for each patient population. Each patient presented to the ED, so triage was considered not matching in those patient encounters with triage output of ‘non-emergent’.

Eligible patients included: 90 HIV, 67 hepatitis C, 11 both HIV and hepatitis C. Full demographics are shown in Table 1A. In regards to ED triage data, a significantly higher percentage of hepatitis C patients (59.7%; 40/67) were found to have an initial diagnosis with emergent criteria than HIV patients (35.6%; 32/90) (Table 1A). Taken together, initial ED presenting symptoms were considered emergent by the symptom checker in 48.8% (77/168) of cases.

**Table 1.** (A) Demographics and triage data, (B) diagnostic accuracy for combined HIV, hepatitis C and both HIV and hepatitis C patients, (C) diagnostic accuracy for HIV only patients, (D) diagnostic accuracy for hepatitis C only patients

(A) Demographics and triage data				
Gender	Female	Male	
	62	106	
	36.9%	63.1%	
Age	Mean	s.d.	
	44.9	12.3	
Disease type	HIV	Hepatitis C	Both
	90	67	11
	53.6%	39.9%	6.6%
Race	White	Black	
	47	121	

Emergent Dx by disease type				
For initial diagnosis	Both	HIV	Hepatitis C	*P*-value
Non-emergent	6	58	27	0.01
Emergent	5	32	40	
Emergent (%)	45.5%	35.6%	59.7%	

(%)	Listed at All	Top1	Top3	Top10	Mean	S.D.
(B) Initial diagnosis: combined (HIV, HEPATITIS C, BOTH)						
Symptomate	8.9	7.1	7.7	8.9	1.5	1.1
Symcat	32.1	11.3	26.9	32.1	2.2	1.4
Isabel	39.3	7.1	16.1	25.6	9.4	9.0
Mayo Clinic	23.8	6.0	13.1	22.0	4.5	4.8
WebMD	36.3	7.1	17.3	28.0	11.8	18.5
(%)	Listed at All	Top1	Top3	Top10	Mean	S.D.
(C) Initial diagnosis: HIV						
Symptomate	5.6	5.6	5.6	5.6	1.0	0.0
Symcat	26.7	6.7	20.0	26.7	2.6	1.5
Isabel	42.4	5.6	17.8	28.9	9.6	9.7
Mayo Clinic	24.4	4.4	10.0	22.2	5.6	5.7
WebMD	35.6	7.8	16.7	26.7	15.1	22.8
(%)	Listed at All	Top1	Top3	Top10	Mean	S.D.
(D) Initial diagnosis: hepatitis C						
Symptomate	11.9	7.5	9.0	11.9	1.9	1.4
Symcat	37.3	16.4	32.8	37.3	1.8	1.3
Isabel	35.8	7.5	13.4	20.9	9.4	7.9
Mayo Clinic	22.4	6.0	14.9	20.9	3.4	3.2
WebMD	32.8	3.0	14.9	23.9	9.8	12.5

s.d., standard deviation; ED, emergency department.

Example of a successful triage for HIV/hepatitis C: A 60-year-old black male with stabbing epigastric pain, radiating to back, fevers, nausea. Physician diagnosis: acute pancreatitis. Symptom checker diagnosis: acute pancreatitis. Symptom checker triage was successfully labelled ‘Emergent’, justifying the patient presenting to the ED and the correct venue.

Example of an unsuccessful triage for HIV/hepatitis C: A 54-year-old black male with epigastric pain, stomach cramping, chills, bloating. Physician diagnosis: Peptic ulcer disease. Symptom checker diagnosis: constipation. Symptom checker triage was labelled ‘Non-emergent’, thereby not in agreement with the patient presenting with reported ‘Emergent’ complaints to the ED.

For HIV and hepatitis C combined, only a mere 6.0–11.3% of the symptom checkers had the initial diagnosis listed as the Top1 diagnosis, with only 8.9–39.3% having the diagnosis listed at all (Table 1B). Significant variations existed for each individual symptom checker, as some appeared more accurate for listing the diagnosis in the top of the differential, *vs.* others more likely to list the diagnosis at all. Data were also shown for HIV and hepatitis C individually, although no direct analysis was made for superiority, as there remained many inconsistencies among the symptom checkers (Table 1C–D). To note, all symptom checkers, whether for combined HIV and hepatitis C, HIV alone or hepatitis C alone had poor diagnostic accuracy in regards to Top1 (<20%), Top3 (<35%), Top10 (<40%), Listed at All (<45%) (Table 1B–D).

This is the first large-scale study to assess real-life, complex patients presenting to the ED in regards to symptom checker diagnostic and triage accuracy, and the first to focus on symptom checkers in the HIV and hepatitis C patient population. Moreover, symptom checker diagnostic capabilities are quite inferior to physician diagnostic capabilities, to a level that may even question their diagnostic potential. Prior analysis utilised a broad array of textbook patient diagnosis with varying results depending on disease severity, commonality and subject matter, but with diagnostic results appearing superior to our patient population and current study [4, 5]. Specifically for diagnostic abilities, our study results likely reflect the nature of real-life, non-textbook cases, in addition to the complex HIV and hepatitis C patient population, something prior studies have not fully addressed [4–7]. Current symptom checker software algorithms may not account for the complex, immunocompromised HIV and hepatitis C patient populations. Our results may suggest the need for general online symptom checker diagnostic algorithms that account for this complex patient subset, as over half of all clinical cases and subsequent symptom checkers do not list the correct diagnosis, albeit in the top 1–10. We do not suggest a separate symptom checker for this patient population, but rather, current symptom checker software to account for complex and dynamic past medical histories and the multifaceted symptoms these patients may encounter.

Key limitations included our lack of direct side-by-side symptom checker comparison to patients not in this population. We also do not account for active disease state, prior treatment status, or antiretroviral or other treatment-specific medication use. Future studies may aim to prospectively compare symptom checkers *vs.* healthcare providers, side-by-side, comparing this patient subset *vs.* the general population. Future studies may also aim to determine symptom checker accuracy based on disease system (i.e. pulmonary *vs.* GI illness). As demand continues to outnumber healthcare access globally, particularly in resource-limited regions, it remains imperative to find non-traditional strategies to mitigate this challenge [8]. Initial ED presenting symptoms were considered emergent by the symptom checker in 48.8% (77/168) of cases. As every patient in this study was in the ED, one would expect a higher proportion of emergent triage from the symptom checkers. However, this may entail symptom checkers not appropriately triaging emergent conditions, or, more likely, may serve as a surrogate for patients inappropriately presenting to the ED when they should have seen their doctor in the clinic or stayed at home for self-care. Regardless, symptom checkers provide the ability of simultaneous and, presumably, cost-effective means of patent screening and triage outside the ED. However, we must consider the ramifications of inappropriate diagnostic and triage advice to already resource-limited EDs.

## Summary

As patients are increasingly utilizing technology prior to seeing a physician, these findings should prompt additional, broader evaluation of symptom checker diagnostic and triage performance in the complex HIV and hepatitis C population. Improvements may start by dynamically revising online symptom checker algorithms to account for more complex medical patients with complex and immunocompromising medical conditions. Current symptom checkers utilise a broad diagnostic cascade and may not account for the disease-specific differential in specific immunocompromising conditions. Future refinement of symptom checkers may benefit from constant analysis of online symptom input queries. Symptom checkers carry the potential for real-time epidemiologic monitoring of patient symptoms, as symptom entries and subsequent symptom checker diagnosis could allow health officials a means to track illnesses in specific patient populations and geographic regions. Symptom checker utilisation could become a surrogate for tracking seasonal and regional diseases in a variety of patient populations. As there remains a significant strain on over-utilised ED resources, symptom checkers could serve as a triage of care, and most specifically, for resource-limited nations globally. The diagnostic and epidemiologic potential for symptom checkers remains vast, however, in order to utilise this potential, further refinement of diagnostic and triage symptom checker algorithms is needed.
